# Role of AI in Competency-Based Medical Education: Using EPA as the Magicbox

**DOI:** 10.34172/aim.31795

**Published:** 2024-11-01

**Authors:** Ali Dabbagh, Firoozeh Madadi, Bagher Larijani

**Affiliations:** ^1^Anesthesiology Research Center, Shahid Modarres Hospital, Shahid Beheshti University of Medical Sciences, Tehran, Iran; ^2^Department of Anesthesiology, School of Medicine, Shahid Modarres Hospital, Shahid Beheshti University of Medical Sciences, Tehran, Iran; ^3^Department of Anesthesiology, School of Medicine, Ayatollah Taleghani Hospital, Shahid Beheshti University of Medical Sciences, Tehran, Iran; ^4^Endocrinology Research Center, Endocrinology and Metabolism Clinical Sciences Institute, Tehran University of Medical Sciences, Tehran, Iran

 Competency-based medical education (CBME) is a modern and quality-based approach to medical education that aims to attain the trainees’ essential professional, behavioral, and scientific capabilities. Implementing entrustable professional activities (EPA) is the cornerstone and fundamental aspect of CBME, which guarantees the formative development of competence in the trainees and the attainment of quality in the required areas of education.

 On the other hand, several researches and studies demonstrate with enough data that artificial intelligence can be used to predict different aspects of neurocognition including attitude and professional behaviors; in other words, artificial intelligence can predict the behaviors, movements, decisions, and reasoning in humans to a great extent; its predictive power, of course, depends on the amount of data and the type of processes.

 Here, we would like to discuss the potential role of artificial intelligence in assessment of medical students in the CBME era.

## Entrustable Professional Activities

 Medical education systems are moving toward CBME, based on the concept that educators should attend to their healthcare profession whenever they become entrustable.^1,2^ Traditional time-based assessment methods are proven to be insufficient for predicting the trustworthiness of residents; hence, medical teachers sought for more reliable assessment tools, one of which is EPA.^3^ Soon after being introduced by Ten Cate in 2005, EPAs gained popularity as an essential component of CBME.^4^ Many institutions have verified EPAs as an assessment tool for medical education including postgraduate medical trainees, undergraduate medical students, many subspecialties, and even junior faculty members.

 To date many, EPAs have been described, and some pilot studies have validated these EPAs. So, the expected levels of entrustment have been defined and many have been even revised^5^; however, the role of using EPA in predicting the professional future of the trainees, especially in the field of medical education decision making, is not yet clear and well-defined.

 A qualified anesthesiologist should be able not only to manage his own stress during critical periods, but also lead a team in this situation. Apparently traditional assessment methods are not adequate to assess such qualifications and EPAs would be promising.^1^ Burkhart and colleagues conducted a survey among anesthesiology programs in USA, Canada, Switzerland, Germany, Austria and the Netherlands. Less than half of respondents already utilized EPAs and even those who developed EPAs had no specific decision-making system based on EPAs.^6^

 Woodworth et al recently published their programmatic system for competency assessment of anesthesiology residency programs in USA. They validated their assessment system which consisted of a set of EPAs, procedural skill assessments, non-technical skill assessments and OSCEs via a mobile application over a period of two years. However, they also confronted some missing assessments.^5^

 A drawback of EPA-based decision making is that the number of EPAs that a trainee in any medical discipline is required to complete is relatively large and based on the content of the curriculum in each discipline; however, this large amount of data per trainee when aggregated within an academic department or beyond, at the level of a college or a university, creates the potential opportunity to draw very diverse and determining conclusions from these data, especially with the help of artificial intelligence.^5^

## Artificial Intelligence and CBME

 Currently a life changing transformation is occurring due to fast development of AI. Expectedly, AI has influenced medical education in many ways as well, one of which is its utilization in student assessment; so, AI can enhance assessment in CBME models in various pathways.^7,8^

 First, some researchers analyzed the narrative feedback to predict those students at risk. Those programs which develop EPAs as an assessment tool, gather a huge amount of data over time. Analyzing the scores and feedback and reconciling them to other forms of assessment may lead to algorithms which may help us find students at risk of failure and intervene.^9,10^

 On the other hand, many articles reported AI’s role in assessment of psychomotor skills, mostly focusing on surgical skills and intubation technique.^11-13^ These technologies might reduce the workload of faculty members, the need for training faculty and also omit human errors. AI can also score the trainees’ function in virtual reality.^1^

 Thinking big, AI can be part of work-based assessment and problem-based learning. By observing trainees in their working environment and combining it with expert feedback and patient outcomes, EPAs might be reliable for making evidence-based decisions about students.^7,14^

## Future Implementation of AI in EPA Assessment

 Since EPA examines different areas based on 6 core competencies, the result of evaluation with the help of EPA is to examine the clinical performance of learners; the final result of which is the improvement of skill, attitude, and knowledge.^15,16^ Therefore, EPA-driven data of each trainee can be used to predict or extrapolate their behavior patterns and, when needed, take steps to improve the behavioral patterns throughout the time when the trainee is under education.

 Therefore, it is possible to use the EPA-obtained data to develop an AI algorithm, which, while predicting clinical performance, simultaneously creates the possibility of improving trainees’ performance. The AI-driven results could also be potentially used to match the graduated people in more appropriate individual positions; either for their professional tasks or any further training position during the future stages.

 Another potential utilization of AI would be supervision in the work environment. In order to utilize EPAs in decision making, programs should collect large data during a long period of time which needs well-educated supervisors and data collecting systems.^14^ AI can be utilized as a supervisor in a digital working environment and validate supervisor’s scoring as well as reducing their workload when adequately trained.

## Conclusion

 Medical education confronts two impressive revolutions:

1. Introducing CBME and EPA as a tool for competency assessment;
2. AI utilization in medical education.

The current disadvantage of EPA is lack of an efficient decision-making system. Recent advances in EPA development and AI can be promising in solving this problem by analyzing the large amount of collected data from both human supervisors and machine learning raters. Using AI to analyze the data retrieved from EPA assessment results might help predict the future professional behavior of the current trainees.
